# Transcultural approach to psychotic episodes. About a case

**DOI:** 10.1192/j.eurpsy.2022.1631

**Published:** 2022-09-01

**Authors:** C. Martín Villarroel, L. Carpio García, L. Santolaya López, G. Belmonte García, M. Sánchez Revuelta, J. Matsuura, E.F. Benavides Rivero

**Affiliations:** Complejo Hospitalario Universitario de Toledo, Psiquiatría, Toledo, Spain

**Keywords:** Psychosis, migration, acculturation, Cultural competence

## Abstract

**Introduction:**

Cultural differences influence understanding and therapeutic adherence of migrant patients, therefore it is very important to acquire cultural competence.

**Objectives:**

The objective of this paper is to study, from the following case, the effect of cultural competence in approach to psychosis in migrant patients.

**Methods:**

A bibliographic search was performed from different database (Pubmed, TripDatabase) about the influence of culture on psychosis and its resolution. A 25-year-old Moroccan man who came to Spain two years ago fleeing his country and suffered violence in different countries until he arrived. He lived on the street until they offered him a sheltered house with other Moroccans. He felt lack of acceptance and loss of his roots. In this context, he developed a first psychotic episode in which he described “the presence of a devil”.

**Results:**

He distrusted antipsychotic treatment and believed “that devil” was still inside him, being convinced that he needed a Muslim healer to expel him. We followed up with the patient and a cultural mediator, better understanding his cultural reality, uprooting and traumas, and he could feel understood and trust us. During the process, he decided to go to the Muslim healer who performed a symbolic rite for which he felt he “expelled the devil”, while accepting antipsychotics. With all this, the psychotic symptoms and their acculturation process improved.

**Conclusions:**

It is very important that psychiatrists have cultural competence to understand the context of migrant patients, and to be able to provide them with the best treatment.

**Disclosure:**

No significant relationships.

